# Role of the CASP-9 Ex5+32 G>A polymorphism in susceptibility to cancer: A meta-analysis

**DOI:** 10.3892/etm.2012.756

**Published:** 2012-10-19

**Authors:** SHI YAN, YONG-ZHI LI, XIN-WANG ZHU, CHUN-LAI LIU, PING WANG, YI-LI LIU

**Affiliations:** Department of Urological Surgery, The Fourth Affiliated Hospital of China Medical University, Shenyang, Liaoning, P.R. China

**Keywords:** caspase-9, polymorphism, cancer, meta-analysis

## Abstract

Failure of apoptosis is one of the hallmarks of cancer. As an execution-phase caspase, caspase-9 plays a crucial role during apoptosis. To examine whether the Ex5+32 G>A (rs1052576) polymorphism in the CASP-9 gene alters cancer risk, we conducted a comprehensive meta-analysis of 7 case-control studies consisting of a total of 1668 cancer cases and 2294 healthy controls. All studies considered, A allele and A allele carriers of Ex5+32 G>A in the CASP-9 gene had significant associations with cancer risk (OR=0.72, 95% CI, 0.58–0.89, P= 0.003; OR= 0.76, 95% CI, 0.63–0.92, P= 0.004; respectively). In the subgroup analysis, we found that the A allele of Ex5+32 G>A was a protective factor for cancer risk in Chinese and American populations (OR=0.60, 95% CI, 0.44–0.81, P<0.001; OR= 0.80, 95% CI, 0.69–0.94, P= 0.005; respectively). Similarly, we also found positive associations between A allele carriers of Ex5+32 G>A and cancer risk in Chinese and American populations (OR=0.63, 95% CI, 0.44–0.90, P= 0.01; OR= 0.78, 95% CI, 0.62–0.98, P=0.03; respectively). In addition, we identified that A allele and A allele carriers of Ex5+32 G>A may decrease the risk of cancer in the Asian population (OR=0.60, 95% CI, 0.44–0.81, P<0.001; OR= 0.63, 95% CI, 0.44–0.90, P= 0.01; respectively). In conclusion, this meta-analysis demonstrated that A allele and A allele carriers of the Ex5+32 G>A polymorphism in the CASP-9 gene may be protective factors for cancer risk.

## Introduction

Apoptosis is an essential mechanism to eliminate unwanted cells during the development and homeostasis of multi cellular organisms (1,2). An imbalance between cell death and proliferation may lead to the incidence of cancer. There are two main apoptotic pathways in humans, the extrinsic pathway and the intrinsic pathway (3). During the apoptotic process, both of the two pathways use the caspase enzyme cascade; the extrinsic pathway utilizes caspases-8 and -10, while the intrinsic pathway employs caspase-9 and the converge to use caspases-3, -6 and -7 as effector caspases, which lead to cell death by nuclear membrane breakdown, DNA fragmentation, chromatin condensation and the formation of apoptotic bodies (4–6).

Caspase-9, an apoptosis-related cysteine peptidase, encoded by the CASP-9 gene, located on chromosome l at 1p36.21, is a member of the caspase (cysteine aspartate protease) family of proteins. It has been shown to be an executioner protein of apoptosis. Caspase-9 plays a central role in the execution-phase of cell apoptosis. Single nucleotide polymorphisms (SNPs) are the most common form of human genetic variation and may contribute to an individual’s susceptibility to cancer (7). In recent years, few studies have been performed to investigate the associations between effector caspases and cancer risk. Lan *et al*(8) found that CASP-9 was significantly associated with a decreased risk for non-Hodgkin lymphoma. Hosgood *et al*(9) found that individuals with the AG and AA genotypes of CASP-9 Ex5+32 G>A experienced a decreased risk of multiple myeloma. However, we are still may unable to reach a consistent conclusion concerning the association between the CASP-9 Ex5+32 G>A (rs1052576) polymorphism and cancer risk according to previous studies. Therefore, we performed a Human Genome Epidemiology (HuGE) review and meta-analysis by including the most recent and relevant articles to identify statistical evidence.

## Materials and methods

### Literature search

We performed an electronic search of the PubMed, Cochrane library, Embase, Web of Science, SpringerLink, CNKI and CBM databases extensively to identify relevant studies available up to May 1, 2012. The search terms included [‘caspase-9’ or ‘CASP-9’ or ‘caspase-9’ (Mesh)] and [‘SNPs’ or ‘SNP’ or ‘polymorphism, genetic’ (Mesh)] and [‘cancer’ or ‘tumor’ or ‘neoplasms’ (Mesh)]. References in the eligible studies or textbooks were also reviewed through a manual search to identify other potentially eligible studies.

### Inclusion and exclusion criteria

The included studies were required to meet the following criteria: i) case-control studies focusing on associations between the CASP-9 Ex5+32 polymorphism and cancer risk; ii) all patients were diagnosed with malignant tumors confirmed by pathological examination of the surgical specimen; iii) the frequencies of alleles or genotypes in case and control groups could be extracted; iv) the publication was in English or Chinese. Studies were excluded when they were: i) not case-control studies concerning CASP-9 Ex5+32 polymorphism and cancer risk; ii) based on incomplete data; iii) irrelevant or overlapping data were reported; iv) meta-analyses, letters, reviews or editorial articles.

### Data extraction

Using a standardized form, data from published studies were extracted independently by two reviewers (S. Yan and Y.-Z. Li) to populate the necessary information. The following information was extracted from each of the articles: first author, year of publication, country, language, ethnicity, study design, source of cases and controls, number of cases and controls, mean age, sample, cancer type, genotype method, allele and genotype frequency, and evidence of Hardy-Weinberg equilibrium (HWE) in controls. In case of conflicting evaluations, an agreement was reached following a discussion with a third reviewer (Y.-L. Liu).

### Quality assessment of included studies

Two reviewers (S. Yan and Y.-Z. Li) independently assessed the quality of papers according to modified STROBE quality score systems (10,11). Forty assessment items related to the quality appraisal were used in this meta-analysis, scores ranging from 0 to 40. Scores of 0–20, 20–30 and 30–40 were defined as low, moderate and high quality, respectively. Disagreement was resolved by discussion.

### Statistical analysis

The odds ratio (OR) and 95% confidence interval (95% CI) were calculated using Review Manager Version 5.1.6 (provided by the Cochrane Collaboration, available at: http://ims.cochrane.org/revman/download) and STATA Version 12.0 (StataCorp, College Station, TX) softwares. Between-study variations and heterogeneities were estimated using Cochran’s Q-statistic (12,13) (P≤0.05 was considered to be a manifestation of statistically significant heterogeneity). We also quantified the effect of heterogeneity by using I^2^ test, which ranges from 0 to 100% and represents the proportion of inter-study variability that can be contributed to heterogeneity rather than by chance. When a significant Q-test (P≤0.05) or I^2^>50% indicated that heterogeneity among studies existed, the random effects model was conducted for meta-analysis. Otherwise, the fixed effects model was used. To establish the effect of heterogeneity on the conclusions of the meta-analyses, subgroup analysis was carried out. We tested whether genotype frequencies of controls were in HWE using the χ^2^ test. Funnel plots are often used to detect publication bias. However, due to limitations caused by varied sample sizes and subjective reviews, Egger’s linear regression test which measures funnel plot asymmetry using a natural logarithm scale of OR was used to evaluate the publication bias (14). When the P-value was <0.1, publication bias was considered significant. All the P-values were two-sided. To ensure the reliability and the accuracy of the results, two reviewers (S. Yan and Y.-Z. Li) populated the data in the statistical software programs independently and obtained the same results.

## Results

### Characteristics of the included studies

**Figure 1:**
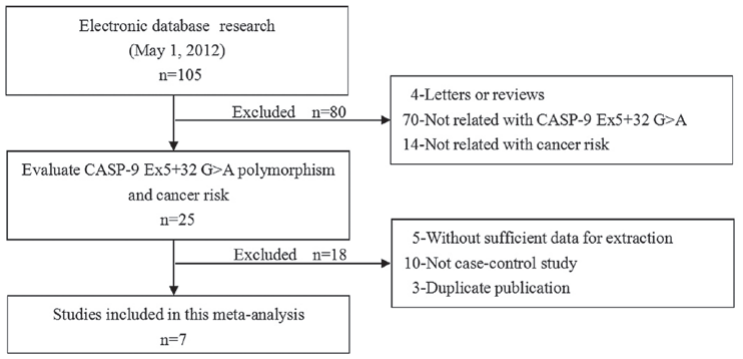
Flow chart shows study selection procedure. Seven case-control studies were included in this meta-analysis.

According to the inclusion criteria, seven studies (8,9,15–19) met the inclusion criteria and were subjected to further examination. The flow chart of the study selection is shown in Fig. 1. In total, 1668 cancer cases and 2294 healthy controls from seven studies were included in the pooled analysis. The publication year of involved studies ranged from 2007 to 2009. Overall, there were six types of cancers studied, including gastric cancer, lymphoma, lung cancer, colon cancer, myeloma and liver cancer. One of these studies was conducted in Russia, two in USA and four studies in China. The HWE test was performed on the genotype distribution of the controls in all included studies; all were in HWE (P>0.05). All quality scores of the included studies were higher than 20 (moderate-high quality). The characteristics and methodological quality of the included studies are summarized in Table I. The genotype distributions of the CASP-9 Ex5+32 G>A polymorphism in the case and control groups are presented in Table II.

### Main results and subgroup analysis

**Figure 2:**
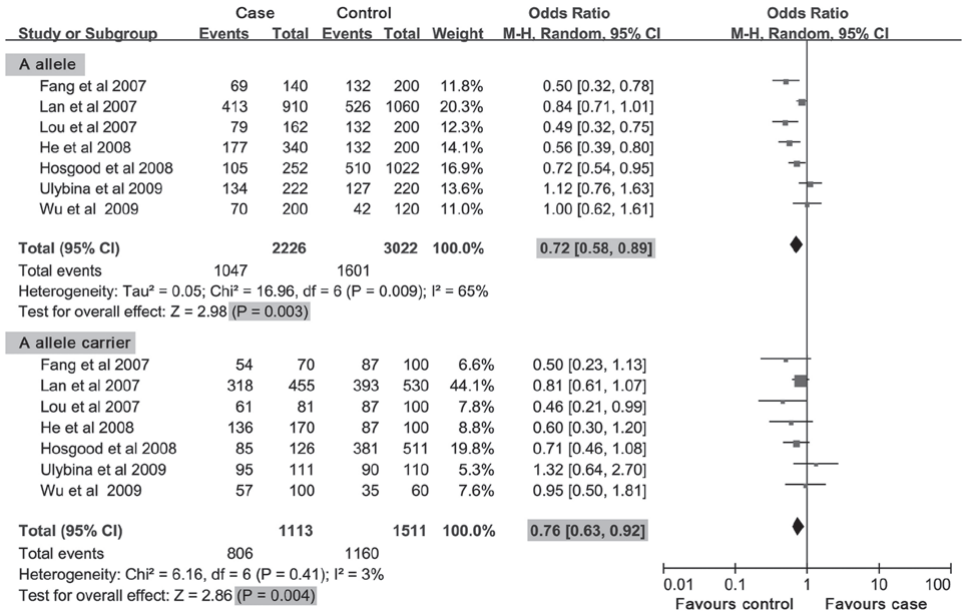
Associations between the CASP-9 Ex5+32 G>A polymorphism and cancer risk.

A summary of the findings of the meta-analysis of the association between CASP-9 Ex5+32 G>A polymorphism and cancer risk is provided in Table III. The meta-analysis results showed that the A allele and A allele carrier of Ex5+32 G>A in the CASP-9 gene had negative associations with cancer risk (OR= 0.72, 95% CI, 0.58–0.89, P= 0.003; OR= 0.76, 95% CI, 0.63–0.92, P= 0.004; respectively) (Fig. 2). In the subgroup analysis by country, we found that the A allele of Ex5+32 G>A was a protective factor for cancer risk in Chinese and American populations (OR= 0.60, 95% CI, 0.44–0.81, P<0.001; OR= 0.80, 95% CI, 0.69–0.94, P=0.005; respectively), but no association was found between the Ex5+32 G>A polymorphism with cancer risk in the Russian population. For the A allele carrier of the Ex5+32 G>A polymorphism, we also found positive associations with cancer risk in Chinese and American populations (OR=0.63, 95% CI, 0.44–0.90, P= 0.01; OR= 0.78, 95% CI, 0.62–0.98, P=0.03; respectively). Unfortunately, there was no significant difference between the Ex5+32 G>A polymorphism with cancer susceptibility in the Russian population. In the subgroup analysis by ethnicity, we found that the A allele and A allele carrier of Ex5+32 G>A in CASP-9 might decrease the risk of cancer in the Asian population (OR=0.60, 95% CI, 0.44–0.81, P<0.001; OR= 0.63, 95% CI, 0.44–0.90, P= 0.01; respectively). However, there were no associations found among A allele and A allele carrier of Ex5+32 G>A in CASP-9 with risk in the Caucasian population (all P>0.05).

### Publication bias

**Figure 3:**
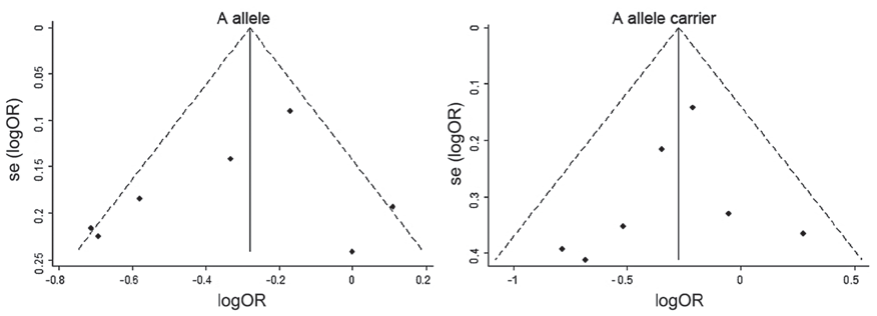
Begger’s funnel plot of publication bias.

Publication bias of the literature was assessed by Begger’s funnel plot and Egger’s linear regression test. Egger’s linear regression test was used to measure the asymmetry of the funnel plot. All graphical funnel plots of the included studies appeared to be symmetrical (Fig. 3). Egger’s test also showed that there was no statistical significance for all evaluations of publication bias (all P>0.05).

## Discussion

Apoptosis is a particular type of programmed cell death which commonly occurs in the developing embryo, in normal healthy adult tissues and in many pathological settings (20). The morphological features of apoptosis include changes in plasma membrane asymmetry and attachment, condensation of cytoplasm, nucleus and internucleosomal cleavage of DNA (21). However, excessive or failed apoptosis is a prominent morphological feature of several human diseases (24). Activation of caspases is of fundamental importance in cell death commitment and hence substantial efforts have been devoted to the understanding of mechanisms that underlie their activation (22,23).

Caspase-9 is a key regulator of apoptosis or programmed cell death, an essential defense mechanism against hyper-proliferation and malignancy. Polymorphic variation in the CASP-9 gene has been reported to influence cancer risk, especially in the Ex5+32 variant. The published studies of an association between the CASP-9 Ex5+32 variant and different cancers have generated inconsistent results. Lou *et al*(16) reported that the rs1052576 which locates in exon 5 of the CASP-9 gene was associated with non-small cell lung cancer. However, a multi-center epidemiological case-control study was not consistent with other previously published data on non-Hodgkin lymphoma (8). This controversy might be due to population and ethnicity of the corresponding studies. In this meta-analysis, including a total of 1668 cancer cases and 2294 healthy controls from seven independent studies, we examined the association of the Ex5+32 G>A polymorphism of the CASP-9 gene with cancer risk. We demonstrated that A allele and A allele carrier in the Ex5+32 G>A polymorphism had negative associations with cancer susceptibility, which showed a protective effect of the CASP-9 gene against cancer development. Ethnicity may influence cancer susceptibility by different genetic backgrounds and environmental exposures through gene-gene and gene-environmental interactions. Subgroup analysis showed that A allele and A allele carrier were protective factors for cancer risk in Chinese and American populations. Contrary to our expectations, we found no association between the Ex5+32 G>A polymorphism and cancer risk in Russian population. In addition, we also identified that A allele and A allele carrier of Ex5+32 G>A might decrease the risk of cancer in the Asian population, but not in the Caucasian population.

Similar to other meta-analyses, a number of limitations of this study should be addressed. First, the relevant research articles are not many and the sample size of this meta-analysis was not large. In addition, some relevant studies could not be included in our analysis due to incomplete raw data. Thirdly, we were not able to address the sources of heterogeneity among all studies. Fourthly, although all cases and controls of each study were well defined with similar inclusion criteria, there may be potential factors that were not taken into account that may have influenced our results. Most important of all, our meta-analysis was based on unadjusted OR estimates since not all published presented adjusted ORs or when they did, the ORs were not adjusted by the same potential confounders, such as ethnicity, gender and geographic distribution. Given these results, additional investigation in these areas is needed, and our conclusions should be interpreted cautiously.

In conclusion, this meta-analysis of seven case-control studies demonstrated that the CASP-9 Ex5+32 G>A polymorphism is involved in the pathogenesis of variant cancer. The A allele, A allele carrier and AA genotype of Ex5+32 G>A polymorphism may be protective factors for cancer risk. As few studies are available in this field current evidence remains limited. Therefore, it is necessary to conduct large studies with adequate methodological quality, properly controlling confounds in order to obtain valid results.

## Figures and Tables

**Table I:** Characteristics of the individual studies in this meta-analysis.

			Number					
Author/(Ref.)	Year	Country	Case	Control	Sample	Genotype method	Cancer type	Quality score
Fang *et al*(15)	2007	China	70	100	Blood	PCR-RFLP	Gastric cancer	23
Lan *et al*(8)	2007	USA	461	535	Blood	DNA sequencing	Lymphoma	22
Lou *et al*(16)	2007	China	81	100	Blood	PCR-RFLP	Lung cancer	24
He *et al*(17)	2008	China	170	100	Blood	PCR-RFLP	Colon cancer	21
Hosgood *et al*(9)	2008	USA	128	516	Blood/Tissue	DNA sequencing	Myeloma	22
Ulybina *et al*(18)	2009	Russia	111	110	Blood	AS-PCR	Lung cancer	24
Wu *et al*(19)	2009	China	647	833	Blood	PCR-RFLP	Liver cancer	28

PCR, polymerase chain reaction; RFLP, restriction fragment length polymorphism.

**Table II:** Genotype distribution of the CASP-9 Ex5+32 G>A polymorphism in the case and control groups.

	Case								Control								HWE test		
Author/(Ref.)	Total	G	A	GG	GA	AA	GA+AA	TA	Total	G	A	GG	GA	AA	GA+AA	TA	χ^2^	P-value	Test
Fang *et al*(15)	70	71	69	16	39	15	54	140	100	68	132	13	42	45	87	200	0.412	0.521	HWE
Lan *et al*(8)	455	497	413	137	223	95	318	910	530	534	526	137	260	133	393	1060	0.188	0.665	HWE
Lou *et al*(16)	81	83	79	20	43	18	61	162	100	68	132	13	42	45	87	200	0.412	0.521	HWE
He *et al*(17)	170	163	177	34	95	41	136	340	100	68	132	13	42	45	87	200	0.412	0.521	HWE
Hosgood *et al*(9)	126	147	105	41	65	20	85	252	511	512	510	130	252	129	381	1022	0.096	0.757	HWE
Ulybina *et al*(18)	111	88	134	16	56	39	95	222	110	93	127	20	53	37	90	220	0.018	0.893	HWE
Wu *et al*(19)	100	130	70	43	44	13	57	200	60	78	42	25	28	7	35	120	0.039	0.843	HWE

HWE, Hardy-Weinberg equilibrium.

**Table III:** Meta-analysis of the association between the Ex5+32 G>A polymorphism and cancer risk.

Comparison	Case n/N	Control n/N	OR (95% CI)	P-value	Effect model
A allele	1047/2226	1601/3022	0.72 (0.58–0.89)	0.003	Random
Subgroup analysis by country					
Chinese	395/842	438/720	0.60 (0.44–0.81)	<0.001	
American	518/1162	1036/2082	0.80 (0.69–0.94)	0.005	
Russian	134/222	127/220	1.12 (0.76–1.63)	0.57	
Subgroup analysis by ethnicity					
Caucasian	652/1384	1163/2302	0.85 (0.70–1.04)	0.11	
Asian	395/842	438/720	0.60 (0.44–0.81)	<0.001	
A allele carrier	806/1113	1160/1511	0.76 (0.63–0.92)	0.004	Random
Subgroup analysis by country					
Chinese	308/421	296/360	0.63 (0.44–0.90)	0.01	
American	403/581	774/1041	0.78 (0.62–0.98)	0.03	
Russian	95/111	90/110	1.32 (0.64–2.70)	0.45	
Subgroup analysis by ethnicity					
Caucasian	498/692	864/1151	0.82 (0.66–1.02)	0.08	
Asian	308/421	296/360	0.63 (0.44–0.90)	0.01	
